# Priming with a Combination of FGF2 and HGF Restores the Impaired Osteogenic Differentiation of Adipose-Derived Stem Cells

**DOI:** 10.3390/cells11132042

**Published:** 2022-06-27

**Authors:** Jeong Seop Park, Doyoung Kim, Hyun Sook Hong

**Affiliations:** 1 Department of Biomedical Science and Technology, Graduate School, Kyung Hee University, Seoul 02447, Korea; godjs@khu.ac.kr (J.S.P.); a0az515@khu.ac.kr (D.K.); 2 East-West Medical Research Institute, Kyung Hee University, Seoul 02447, Korea; 3 Kyung Hee Institute of Regenerative Medicine (KIRM), Medical Science Research Institute, Kyung Hee University Medical Center, Seoul 02447, Korea

**Keywords:** adipose-derived stem cell, paracrine potential, osteogenic differentiation, hepatocyte growth factor, fibroblast growth factor 2

## Abstract

Classical aging-associated diseases include osteoporosis, diabetes, hypertension, and arthritis. Osteoporosis causes the bone to become brittle, increasing fracture risk. Among the various treatments for fractures, stem cell transplantation is currently in the spotlight. Poor paracrine/differentiation capacity, owing to donor age or clinical history, limits efficacy. Lower levels of fibroblast growth factor 2 (FGF2) and hepatocyte growth factor (HGF) are involved in cell repopulation, angiogenesis, and bone formation in the elderly ADSCs (ADSC-E) than in the young ADSCs (ADSC-Y). Here, we study the effect of FGF2/HGF priming on the osteogenic potential of ADSC-E, determined by calcium deposition in vitro and ectopic bone formation in vivo. Age-induced FGF2/HGF deficiency was confirmed in ADSCs, and their supplementation enhanced the osteogenic differentiation ability of ADSC-E. Priming with FGF2/HGF caused an early shift of expression of osteogenic markers, including Runt-related transcription factor 2 (Runx-2), osterix, and alkaline phosphatase (ALP) during osteogenic differentiation. FGF2/HGF priming also created an environment favorable to osteogenesis by facilitating the secretion of bone morphogenetic protein 2 (BMP-2) and vascular endothelial growth factor (VEGF). Bone tissue of ADSC-E origin was observed in mice transplanted with FGF/HGF-primed ADSC-E. Collectively, FGF2/HGF priming could enhance the bone-forming capacity in ADSC-E. Therefore, growth factor-mediated cellular priming can enhance ADSC differentiation in bone diseases and thus contributes to the increased efficacy in vivo.

## 1. Introduction

Fractures are one of the leading causes of disability and death, incurring enormous socio-economic costs [1]. In Korea, the 1-year cumulative mortality rate in patients aged ≥50 years after hip fractures was 16.0% (4547/28,426) [2]. Globally, more than nine million people suffer from bone fractures every year, and the incidence tends to increase with age [1,2,3]. Particularly, underlying conditions such as osteoporosis can aggravate the pathogenesis in skeletal tissue. Osteoporotic fractures were reported to elevate subsequent fracture risk [3,4,5]. Hence, therapeutic strategies to prevent fatality due to bone loss and fracture are undoubtedly required for an aging society. Clinical treatment of fractures employs surgical immobilization, ultrasound therapy, and bone grafts, but these are limited by their underwhelming regeneration capacity, risk of infection, and side effects [6,7,8,9]. Therefore, the development of novel therapies for bone regeneration is the need of the hour.

Currently, stem cell therapy has emerged as an attractive choice for bone regeneration [10]. Stem cells possess the ability of self-renewal with multi-differentiation and paracrine potential [11,12,13]. Plenty of clinical research has attempted to apply stem cell therapy to incurable diseases and has proved its therapeutic effect in vivo. The application of stem cells for bone disorders has been attempted by the transplantation of stem cells supplemented with scaffold or growth factors [13,14,15], confirming stem cell-mediated bone-forming capacity.

Adult stem cells available for transplantation reside in various tissues, including bone marrow, adipose tissue, umbilical cord blood, and dental tissues. Bone marrow stem cell (BMSC) is regarded as the primitive stem cell [15,16,17]. Notably, the osteogenic potential of BMSCs was attested in many studies [18,19,20], which motivated BMSC utilization to alleviate skeletal defects in the clinic. However, the efficacy of BMSCs is considerably influenced by disease conditions and the donor age [21,22]. BMSCs from aged patients showed low cellular activity and survival during ex vivo culture [23]; therefore, it is challenging to use the patient’s BMSCs for autologous transplantation. Nowadays, an adipose-derived stem cell (ADSC) commands attention as an alternative to BMSCs. ADSCs are easily isolated from adipose tissue with low donor-site morbidity and are free from ethical controversy and immunogenic problems [24,25]. Importantly, the cellular activity of ADSCs is reported to be rarely altered by the disease condition compared to BMSCs [26]. These merits place ADSCs in the spotlight of pharmacological and clinical developments.

Various preclinical studies have induced ADSC differentiation into bone tissue [27,28,29]. Nevertheless, it should be noted that ADSC activity could be affected by age or disease, even though its impact is less than in BMSCs [30,31,32,33]. In practice, ADSCs from the elderly show relatively low differentiation potential, slow proliferation, insufficient paracrine ability, and rapid senescence compared to the young [30,33,34]. These data suggest that the autologous application of ADSCs the elderly requires a confident strategy to improve the cellular activity of ADSCs before transplantation. If not, transplanted ADSCs would have a diminished therapeutic impact with undesired effects in vivo. To enhance the therapeutic capability of ADSCs, several methods, such as pre-conditioning by cytokines, genetic manipulation, physical stimuli, extracellular vesicle, and cultures with three-dimensional aggregates have been employed so far [35,36,37,38,39]. However, the problems of biodegradability, anxiety about genetic manipulation, side effects, and unsatisfactory therapeutic impact are still significant challenges [40,41,42]. 

To improve the differentiation activity of ADSCs that is impaired by age or disease, reversal of the secretory deficiency is expected to restore its cellular activity fundamentally. Cellular differentiation occurs through the interplay of cytokine/growth factors in an autocrine/paracrine manner. For enhanced differentiation, ADSCs should mount an appropriate cellular response to extracellular stimuli to activate differentiation. However, aging/senescence impairs paracrine potential and receptor occupancy of ADSC, creating a deficiency of essential secretory factors [33,43]. Accordingly, the modulation of the secretory condition of ADSCs is surmised to be decisive for its differentiation potential.

Primary molecular regulators for osteogenesis are fibroblast growth factor 2 (FGF2), transforming growth factor beta-1 (TGF-β1), bone morphogenic protein-2 (BMP-2), and vascular endothelial growth factor (VEGF) [44,45,46] and they are spontaneously produced in ADSCs and activate specific signaling pathways upon osteogenic stimulation. Of these, FGF2 is a pleiotropic signaling molecule involved in angiogenesis, cell growth, and tissue repair [44,47]. Additionally, FGF2 was found to promote bone formation, accompanied by the enhanced secretion of VEGF [48] and BMP-2 [49]. Importantly, FGF-2 expression is reduced with aging in various tissues [50,51]. TGF-β1 and VEGF have also been reported to enhance bone formation, but their levels are relatively unaffected by aging [33,52,53].

The hepatocyte growth factor (HGF) is a versatile growth factor controlling organogenesis, tissue repair, and bone remodeling via phosphorylation of C-met [54]. HGF is reported to improve bone regeneration via the production of BMP-2 [55,56]. Moreover, HGF induces VEGF expression, aiding bone formation through its angiogenic properties [57]. The secretion of HGF is also reduced by cellular senescence, disease, or donor age [33]. 

Based on the insufficient secretion induced by age, minimal manipulation of ADSCs with growth factors is estimated to improve osteogenesis. In this study, ADSC-E and ADSC-Y were cultured ex vivo, and their levels of paracrine factors related to osteogenesis were quantitatively compared. Then, a method to restore the osteogenic potential of ADSC-E was established by supplementation of growth factors. The effect of growth factor-priming on ADSCs was determined by early osteogenic marker expression, calcium production in vitro, and bone-forming capacity in vivo.

## 2. Materials and Methods

### 2.1. Cell Culture

The elderly’s adipose tissues were provided by the Kyung Hee University Medical Center [Seoul, Korea; (IRB# 2016-12-022, donor: 8, 2021-01-011, donor: 20)] with the written agreement of the donors. Adipose tissues immediately harvested from donors aged 50–70 were washed in PBS with 5% Penicillin/streptomycin (Welgene, Daegu, Korea). After washing, the tissues were enzymatically digested using 1% collagenase I for 1 h at 37 °C. The digestion was stopped by adding the same volume of FBS. After centrifugation, the stromal vascular fraction was filtered through a cell strainer (70 μm, Corning, NY, USA) to remove debris and centrifuged at 1500 rpm for 5 min at 4 °C to obtain ADSC pellets. Collected ADSCs were resuspended in α-MEM with 10% FBS and 1% penicillin and streptomycin. ADSCs from healthy young individuals were purchased from ScienCell Research Laboratories (Carlsbad, CA, age: 20–29 years) and cultured. All ADSCs were cultured in a 37 °C, 5% CO2 incubator, and the culture medium was changed every other day. During all experiments, ADSCs between passages 3–5 was used.

### 2.2. Osteogenic Induction and Growth Factor Treatment

The ADSCs were seeded into 6-well plates (5 × 10^4^ cells/ well). When cell confluency was approximately 80–90%, the culture media was replaced with Stempro osteogenesis differentiation media (Gibco, Grand Island, NY, USA) and cultured for 20 days. During osteogenic induction, the ADSCs were primed with 1 or 5 ng/mL of FGF2 (R&D systems, Minneapolis, MN, USA) or 10 or 50 ng/mL HGF (R&D systems, Minneapolis, MN, USA) or their combination for 1, 3, or 6 days after osteogenic induction. After completion of priming of FGF2 and/or HGF, the ADSCs were maintained in osteogenesis differentiation media for 20 days. On the 20th day after osteogenic induction, cells were fixed with 3.7% formaldehyde (Sigma-Aldrich, ST. Louis, MO, USA) and stained with 2% Alizarin red S solution (Sigma-Aldrich, ST. Louis, MO, USA) for 10 min to visualize calcium deposition. Alizarin red S was eluted using 10% cetylpyridinium chloride solution (Sigma-Aldrich, ST. Louis, MO, USA), and calcium deposition was quantified as the absorbance value at a wavelength of 560 nm (Molecular Devices, Sunnyvale, CA, USA).

### 2.3. Western Blot

ADSCs at 0, 1, 3, and 6 days after osteogenic induction were washed with PBS and lysed with 1X lysis buffer (Cell Signaling Technology, Danvers, MA, USA). The supernatants were collected by centrifugation at 12,000 rpm for 20 min at 4 °C. The protein concentration was determined by the bicinchoninic acid (BCA) assay (Thermo Fisher, Rockford, IL, USA). The lysates were denatured and electrophoresed using SDS-PAGE and transferred to a nitrocellulose membrane. After blocking with 5% skim milk, the membranes were incubated with the primary antibodies for C-Met and P-Met, FGF2, Runx-2 (Cell Signaling Technology, Danvers, MA, USA), fibroblast growth factor receptor 2 (FGFR2), Osterix, alkaline phosphatase (ALP), and glyceraldehyde 3-phosphate dehydrogenase (GAPDH) (Abcam, Cambridge, UK), followed by anti-IgG horseradish peroxidase-conjugated secondary antibody (Bio-rad, Hercules, CA, USA). The blots were developed by adding ECL (Dogen Bio, Seoul, Korea); chemiluminescence was visualized with an Amersham imager 600 (GE Healthcare, Buckinghamshire, UK). The expression level was quantified using the ImageJ program (Version 1.53e, National Institutes of Health, Bethesda, MD, USA).

### 2.4. Animals Model

Six-week-old Balb/c nude mice (20–22 g, male) were purchased from Daehan Bio Link (Seoul, Korea). The mice were housed under a 12 h light/dark illumination cycle in an experimental animal room and permitted to adapt for 7 d before the commencement of the experiments. All animals received standard chow and water ad libitum, and this study was approved by the Ethical Committees for Experimental Animals of Kyung Hee University Hospital with the approval number KHMC-IACUC-20-008-01, 02.

### 2.5. Ectopic Bone Formation and Histological Analysis

The ADSCs were primed for 3 and 6 days under osteogenic induction with FGF2, HGF, and FGF2/HGF, respectively, and then, 2 × 10^6^ ADSCs were mixed with 40 mg of hydroxyapatite/beta-tricalcium phosphate (HA/β-TCP) ceramic powder (Biomatlante, Vigneux-de-Bretagne, France). The ADSC-HA/β-TCP mixture was incubated at 37 °C for 2 h and implanted subcutaneously into the dorsal region of Balb/c nude mice. 12 weeks later, the implants were harvested and fixed in 3.7% formaldehyde. The samples were decalcified in 0.2 M EDTA (PH 7.2~7.4) for 2 weeks and embedded in paraffin. 

The paraffin-embedded sample was sectioned to a 5-μm thickness. After deparaffinization and hydration, hematoxylin and eosin (H&E) staining was performed. To detect the transplanted human ADSCs, samples were treated with an antibody for human osteocalcin and incubated with a biotin-conjugated secondary antibody. Enzyme-substrate reaction was carried out with ABC reagent solution. The stained area was visualized with Nova RED (Vector Laboratories, Burlingame, CA, USA), and counterstaining was completed with hematoxylin.

### 2.6. Statistical Analysis

All data are presented as the mean ± standard deviation. Statistical analyses were performed using GraphPad Prism (GraphPad Software, version 5.01, San Diego, CA, USA). Differences were considered statistically significant at *p* < 0.05 and were interpreted as follows: * *p* < 0.05, ** *p* < 0.01, *** *p* < 0.001. Statistical analysis was performed using an unpaired two-tailed Student’s *t*-test.

## 3. Results

### 3.1. ADSCs with Weak Osteogenic Potential Is Deficient in Paracrine Factors under Normal Physiological Conditions

**Figure 1:**
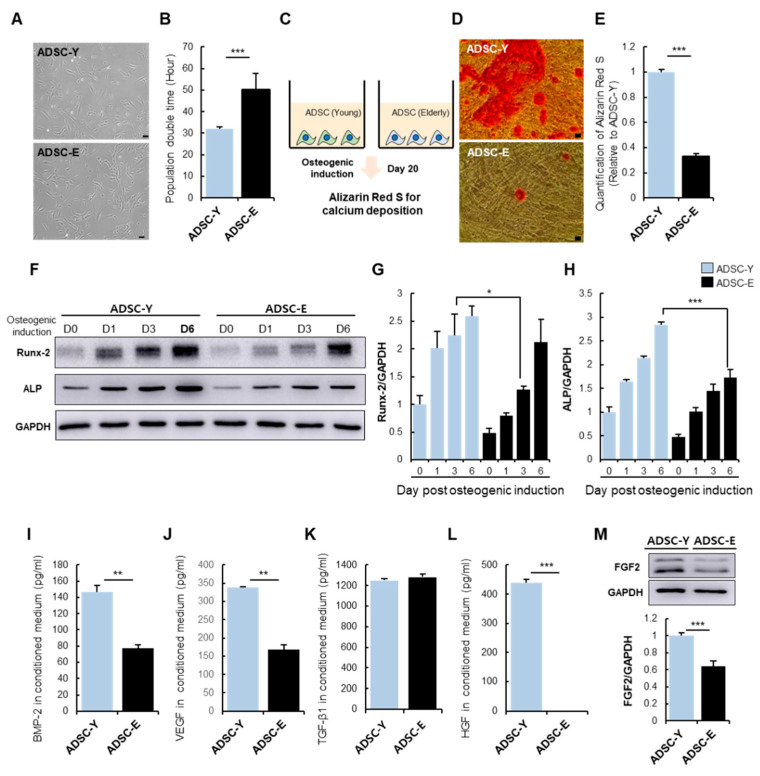
Effect of age on the osteogenic/paracrine potential of ADSCs.(**A**) Comparison of cellular morphology of ADSC-Y and ADSC-E. Scale bar, 100 μm. (**B**) Population doubling time was analyzed. (**C**) Experimental scheme for comparative analysis of osteogenesis in ADSC-Y and ADSC-E. (**D**) Representative images for Alizarin Red S staining of ADSC-Y and ADSC-E after osteogenic induction for 20 days. Scale bar, 100 μm. (**E**) Alizarin Red was quantified with 10% (*w/v*) cetylpyridinium chloride. (**F**) ADSC was lysed at 0, 1, 3, and 6 days after osteogenic induction to analyze osteogenic markers. (**G**,**H**) Expression of Runx-2 and ALP was confirmed by western blot. (**I**–**L**) The level of BMP-2, VEGF, TGF-β1, and HGF in the conditioned medium of ADSC-Y and ADSC-E was quantified by ELISA. (**M**) Protein level of FGF-2 was determined by western blot and quantified by ImageJ. Results are shown as the mean ± SD of three replicate wells for each group. * *p* < 0.05, ** *p* < 0.01, and *** *p* < 0.001.

For comparative analysis of the cellular activity of young ADSCs (ADSC-Y) and elderly ADSCs (ADSC-E), ADSCs were isolated and cultured from the young and elderly, respectively. Differences in cellular morphology (Figure 1A) and surface maker expression (Table S1) between ADSC-Y and ADSC-E were rarely observed. However, ADSC-Y had a doubling time of approximately 30 h, whereas ADSC-E had doubled in 50 h (Figure 1B). ADSCs were cultured in osteoinductive media for 20 days to evaluate the osteogenic potential, after which alizarin Red S staining was performed to examine calcium deposition (Figure 1C,D). ADSC-Y could differentiate into osteoblasts with high calcium deposition, whereas ADSC-E seldom differentiated into osteoblasts under osteoinductive conditions (Figure 1D,E). This result is consistent with previous studies [33].

Osteogenic differentiation progresses via the activation of Runx-2, a major transcriptional regulator of early osteogenesis. Runx-2 expression was lower in ADSC-E than ADSC-Y during the early phase of osteogenic induction (Figure 1F,G). A higher level of ALP expression was also detected in ADSC-Y (Figure 1F–H). This phenomenon might be related to the loss of osteogenic potential of ADSC-E.

Runx-2 activation involves signaling molecules such as TGF-β, FGF, and BMP-2 [46,47,49]. Next, to check the paracrine potential of ADSC-Y and ADSC-E, the secretion of osteogenesis-enhancing growth factors, including BMP-2, VEGF, TGF-β1, and HGF, was evaluated. The levels of BMP-2 and VEGF were significantly higher in ADSC-Y than in ADSC-E (Figure 1I,J). Unexpectedly, the production of TGF-β1 in ADSCs was not affected by age and osteogenic capacity (Figure 1K). The difference in HGF secretion was considerable between ADSC-Y and ADSC-E, suggesting that secretion of HGF may be deeply related to the osteogenic potential of ADSCs (Figure 1L). The levels of FGF-2, a representative factor that promotes bone formation, has been reported to decrease in the skin and muscle with age [50,51], which is also observed in ADSCs. ADSC-E had a low expression level of FGF2 compared to ADSC-Y (Figure 1M).

This result suggests that age affects cell repopulation rate and differentiation potential, which may be attributed to the alteration of paracrine factors.

### 3.2. ADSCs with the Deficient Osteogenic Potential Shows Impaired Paracrine Action in Response to Osteogenic Stimulus

To ascertain the relation between osteogenic potential and paracrine factors, the kinetics of osteogenesis-related paracrine factors during osteogenic induction was examined in ADSC-Y and ADSC-E, respectively.

**Figure 2:**
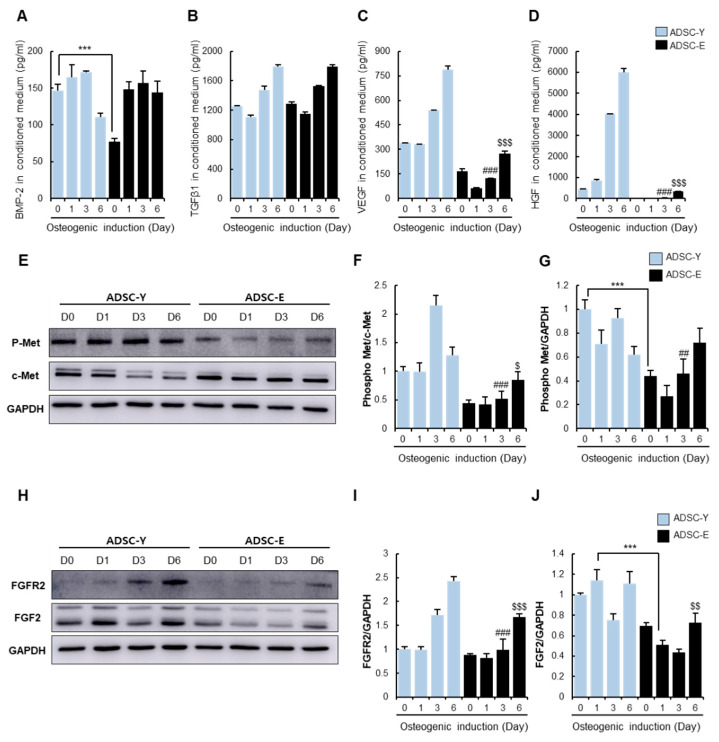
The analysis of the expression pattern of osteogenic factors in ADSC-Y and ADSC-E under osteogenic induction. ADSC-Y and ADSC-E were cultured in osteogenic media, respectively, and its conditioned medium was collected at 0, 1, 3, and 6 days after osteogenic induction. (**A**–**D**) Levels of BMP-2, TGF-β1, VEGF, and HGF levels in a conditioned medium were examined by ELISA. (**E**–**G**) P-Met and C-Met expression levels in ADSCs were analyzed by western blot and quantified relatively to C-Met or GAPDH. (**H**–**J**) FGFR2 and FGF2 proteins in ADSCs were determined by western blotting and quantified relatively. Results are shown as the mean ± SD of three replicate wells for each group. *** *p* < 0.001, ## *p* < 0.01, ### *p* < 0.001 vs. Day 3 of ADSC-Y and $ *p* < 0.05, $$ *p* < 0.01, $$$ *p* < 0.001 vs. Day 6 of ADSC-Y.

As shown in Figure 1I, osteogenic stimuli elevated BMP-2 concentration in ADSC-E, similar to that of ADSC-Y (Figure 2A). Therefore, osteogenic stimuli could resolve the deficiency of BMP-2 in ADSC-E. The level of TGF-β1 was sustained in a similar pattern in both ADSC-Y and ADSC-E in osteogenic conditions (Figure 2B). VEGF secretion was gradually elevated in ADSC-Y, while its level almost remained unchanged in ADSC-E for six days (Figure 2C). HGF levels were constantly elevated post osteogenic induction, but HGF in ADSC-E was too low to be detected (Figure 2D). This phenomenon was repeatedly observed by cytokine array (Figure S1). HGF binds to the receptor c-Met and autophosphorylates it to transduce various signaling pathways (5). As predicted, ADSC-Y with active HGF secretion showed higher phosphorylation levels of C-Met, compared to ADSC-E (Figure 2E–G). The comparative analysis revealed that FGF2 and FGF2R expression is maintained higher in ADSC-Y than in ADSC-E, indicating a possible dynamic response of FGF2 signaling in ADSC-Y rather than ADSC-E (Figure 2H–J).

Considering the difference in paracrine potentials from ADSC-Y and ADSC-E during the initial stage of osteoinduction, BMP-2 or TGF-β is not anticipated to be directly related to the loss of osteogenic potential of ADSC-E because of the non-significant difference between ADSC-Y and ADSC-E. It can be surmised that the scarcity of HGF, FGF2, or VEGF directly impinges on the impaired osteogenic potential of ADSC-E.

### 3.3. FGF2/HGF Priming Promotes Osteogenic Differentiation of ADSCs

This study discovered that VEGF, HGF, and FGF2 are insufficient in ADSCs with low osteogenic activity. To improve the differentiation ability of ADSC-E, the deficient growth factor would need to be restored in ADSC-E. Thus, VEGF, HGF, and FGF2 were considered potential candidates to enhance the osteogenic activity of ADSC-E (Figure 2). 

FGF2 and HGF can elevate BMP-2 and VEGF in diverse cell types [48,55,56,57], and thus, FGF2 or HGF treatment was anticipated to create a VEGF-enriched condition in ADSC-E. It was ultimately examined whether supplementation of FGF2 and/or HGF in ADSC-E can restore osteogenic potential or not. 

Modulating the early osteogenic protein expression, including Runx-2 and ALP, is expected to be decisive for osteogenic potential. However, the earliest time window to promote osteogenic protein expression during osteogenic induction is unknown and the development of stem cells to pre-osteoblast with increased Runx-2 was merely known to take 6–7 days in vitro [58]. 

**Figure 3:**
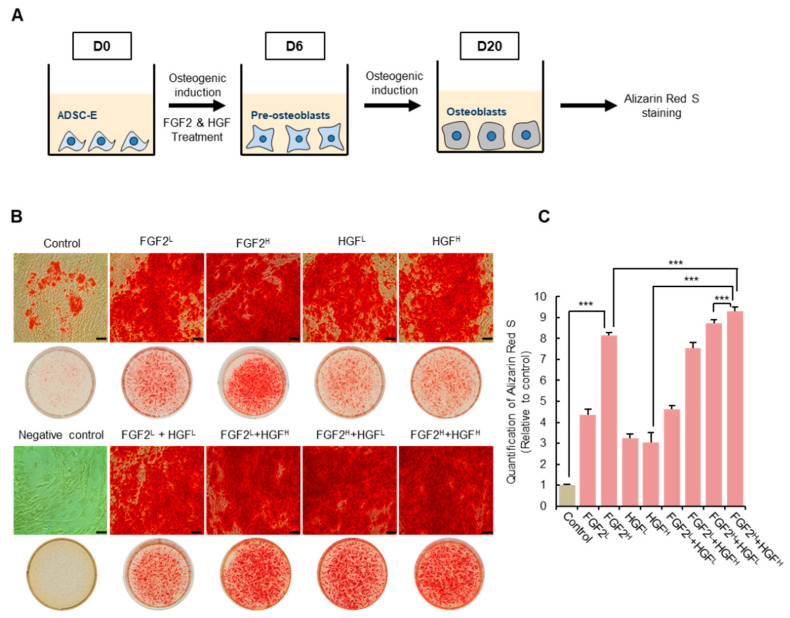
FGF-2/HGF priming enhances the osteogenic potential of ADSC-E. (**A**) Experimental schedule to treat ADSC-E with FGF2 and/or HGF during the osteo-inductive condition. (**B**,**C**) Representative images of Alizarin Red S staining of ADSC-E in each condition and the quantification of calcium deposition. Scale bar, 100 μm. Results are shown as the mean ± SD of four replicate wells for each group. *** *p* < 0.001. FGF2^L^: 1 ng/mL, FGF2^H^: 5 ng/mL, HGF^L^: 10 ng/mL, HGF^H^: 50 ng/mL.

To clarify the optimal time to stimulate the differentiation of ADSC-E, ADSC-E was treated with FGF2 and/or HGF (FGF2: 1 or 5 ng/mL; HGF: 10 or 50 ng/mL) for 3 or 6 days after osteogenic induction. Thereafter, ADSCs were maintained in osteogenic condition without FGF2 and/or HGF until 20 days (Figure 3A).

FGF2 or HGF priming for six days distinctly enhanced the osteogenic potential of ADSC-E (Figure 3B). Notably, the effect of FGF-2 was rarely observed in ADSC-Y, but a prominent effect was shown in ADSC-E (Figure S2), indicating the significance of FGF2 supplementation for osteogenic potential. FGF2/HGF combination could considerably recover the osteogenic activity of ADSC-E, similar to ADSC-Y (Figure 3 and Figure S3). To determine the precise effect of each condition, Alizarin Red S corresponding to calcium deposition was quantified. A remarkable improvement was observed when primed with a combination of FGF2 and HGF, rather than FGF2 or HGF alone (Figure 3C). The effect of FGF2 or HGF was also determined to be the most effective in 5 ng/mL FGF2 and 50 ng/mL HGF. This dose was used for further experiments.

Treatment with FGF2 and/or HGF for three days post osteogenic induction was not enough to restore ADSC-E’s osteogenic activity despite the obvious effect of FGF2 and/or HGF (Figure S4). 

These results demonstrated that the initial supplementation of FGF2 and/or HGF for six days could enhance the osteogenic capacity of ADSC-E.

### 3.4. FGF2/HGF Priming-Mediated Osteogenic Improvement Occurs by Modulation of Early Osteogenic Gene Expression

**Figure 4:**
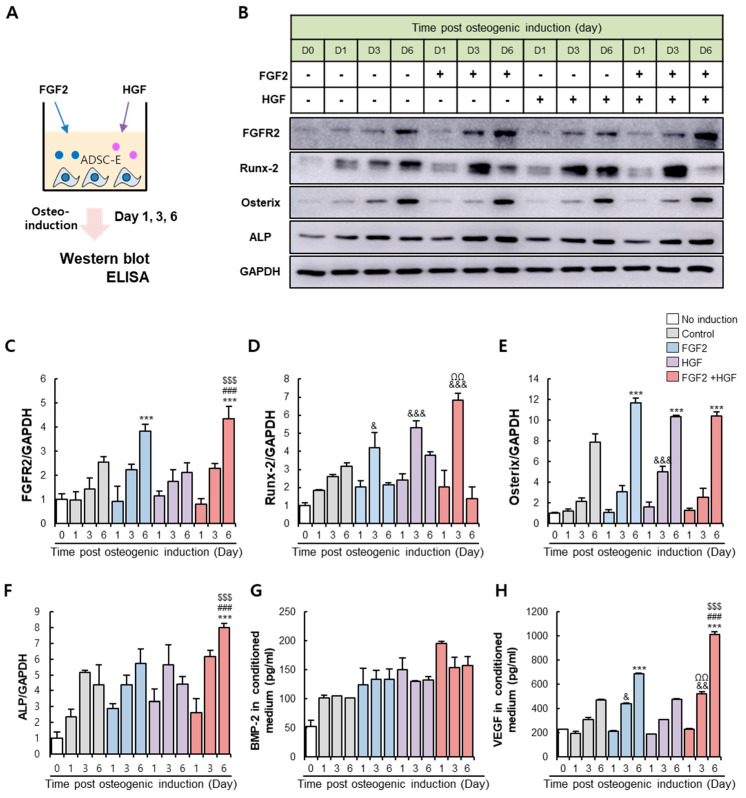
FGF-2 and HGF priming regulate the expression of early osteogenic markers in ADSC-E under osteogenic conditions. (**A**) Experimental scheme for analyzing the effect of FGF-2 and/or HGF priming on osteogenic markers on ADSC-E. (**B**–**F**) FGFR2, Runx-2, Osterix, and ALP levels in ADSC-E were examined by western blot. (**G**,**H**) Quantification of BMP-2 and VEGF in a conditioned medium was performed by ELISA. Results are shown as the mean ± SD of five replicate wells for each group. & *p* < 0.05, && *p* < 0.01, &&& *p* < 0.001 vs. Day 3 of control, ΩΩ *p* < 0.01 vs. Day 3 of HGF-primed ADSC-E, *** *p* < 0.001 vs. Day 6 of control, ### *p* < 0.001 vs. Day 6 of FGF2-primed ADSC-E, $$$ *p* < 0.001 vs. Day 6 of HGF-primed ADSC-E.

Stimulation of ADSC-E with FGF2 and/or HGF for six days suitably enhanced osteogenic differentiation of ADSC-E, suggesting the importance of the cellular event that occurred for six days in the presence of FGF2 and/or HGF. Next, osteogenic induction was applied to ADSC-E, and the expression pattern of the representative osteogenic proteins was monitored at 1, 3, and 6 days after osteogenic induction (Figure 4A).

Under the osteogenic condition, the expression of FGFR2 increased in a time-dependent manner, which was pronounced in FGF2 or a combination of FGF2/HGF (Figure 4B,C). Runx-2 showed a time-dependent increase in the non-treated control group, consistent with previous reports [58]. However, FGF2 and/or HGF treatment facilitated Runx-2 expression and shifted its peak to day three. The expression level of Runx-2 was highest when in a combination of FGF2/HGF treatment (Figure 4B,D). Osterix and ALP expressions were slightly changed by FGF2 and/or HGF compared to the control (Figure 4B,E,F). Based on the protein expression profile, FGF2 and/or HGF were predicted to make ADSC-E enter the phase of immature pre-osteoblast from ADSC-E earlier compared to non-treated ADSC-E control.

Among secretory factors with differing basal levels in ADSC-E and ADSC-Y, BMP-2 and VEGF were evaluated. Compared to the control, BMP-2 was sustained at a higher level in the presence of FGF2 and HGF. FGF2 or HGF treatment could not make a significant difference among groups. The combination of FGF2/HGF showed the highest concentration from day one post-treatment (Figure 4G). VEGF secretion was elevated in osteoinduction, and its concentration was exceedingly not affected by FGF2 or HGF priming. However, a combination of FGF2/HGF unambiguously enriched VEGF in ADSC-E (Figure 4H).

FGF2/HGF priming for six days under osteogenic induction promoted osteogenic protein expression, accompanied by osteogenesis-favored paracrine condition. This environment is expected to provide the cellular environment to improve the osteogenic potential in ADSC-E during an early phase of the osteogenic induction.

### 3.5. FGF2/HGF Priming Enhances the Bone-Forming Capacity of ADSCs In Vivo

**Figure 5:**
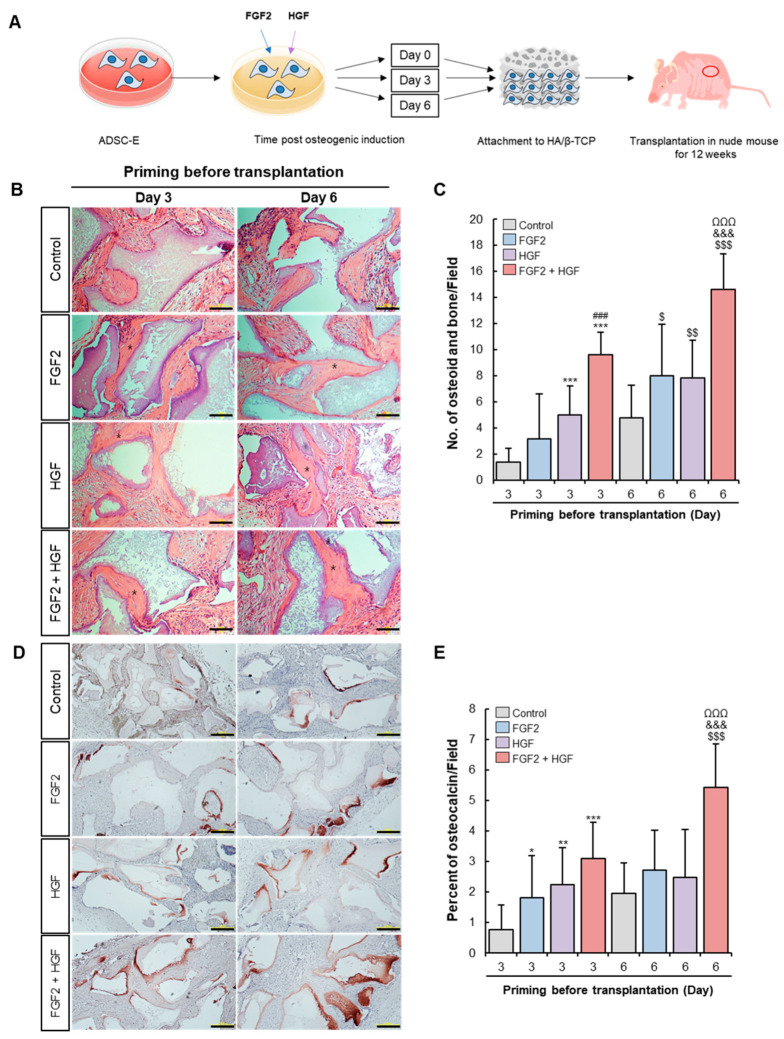
FGF-2 and HGF priming improve the bone forming capacity of ADSC-E in vivo. (**A**) Experimental design for cell transplantation of FGF-2 and/or HGF-primed ADSC-E. (**B**,**C**) H&E staining for the complex of transplanted cells and bone particles. Scale bar, 200 μm. (**D**,**E**) Human osteocalcin stained immunohistochemically was quantified with ImageJ. Scale bar, 200 μm. Results are shown as the mean ± SD of three replicate wells for each group. * *p* < 0.05, ** *p* < 0.01, *** *p* < 0.001 vs. Day 3 of control, ### *p* < 0.001 vs. Day 3 of HGF-primed ADSC-E, $ *p* < 0.05, $$ *p* < 0.01, $$$ *p* < 0.001 vs. Day 6 of control, &&& *p* < 0.001 vs. Day 6 of HGF-primed ADSC-E and ΩΩΩ *p* < 0.001 vs Day 3 of FGF2/HGF-primed ADSC-E.

To evaluate the osteogenic capacity of FGF2 and/or HGF-primed ADSCs in vivo, ADSC-E was cultured in the presence of FGF2 and/or HGF in osteogenic media. Three or six days later, ADSC-E was mixed with HA/β-TCP and then transplanted into nude mice to allow in vivo differentiation (Figure 5A). H&E staining showed the formation of osteoid and newly formed bone. FGF2 and/or HGF priming could enhance bone-forming capacity compared to non-treated control, and the combination of FGF2/HGF was superior to FGF2- or HGF-primed cells for both day 3 and day 6. The most osteogenic ability was observed when primed with a combination of FGF2/HGF for 6 days (Figure 5B,C).

Osteocalcin is synthesized by osteoblast and is a major bone formation marker. To track bone tissue of human origin, human-specific osteocalcin expression was determined by immunohistochemistry. A small area positive for osteocalcin was observed in the control group; its level increased upon FGF2 and/or HGF priming (Figure 5D). Quantification of the osteocalcin-stained area proves that a considerable expression of osteocalcin is observed upon priming with a combination of FGF2/HGF for 6 days only. That is, the combination of FGF2/HGF works best for ADSC-E and could facilitate the differentiation into osteoblast in vivo. 

Collectively, these results corroborate that early priming of combination of FGF2/HGF is required for the improvement of osteogenic potential of ADSC-E in vivo.

## 4. Discussion

The need for novel treatments for bone regeneration of the elderly has become a major challenge in the clinical field. To address this issue, tissue engineering with stem cells has been attempted, where ADSCs or BMSCs are mainly employed as the reparative cellular source. The therapeutic ability of BMSCs is affected by donor disease or age. BMSCs show a low paracrine/differentiation potential in cases of the elderly with the disease [23,59]. In contrast to BMSCs, the application of ADSCs has advantages, including similar features to BMSCs, easy access to the tissue, and rapid proliferation rate in vitro. Thus, many clinical/non-clinical studies conducted with ADSCs prove the efficacy of ADSCs in critical diseases such as bone, skin, or cartilage defects. However, aged patients primarily need stem cell transplantation: age substantially affects ADSC activity, leading to increased doubling time, insufficient paracrine factors, and decreased differentiation potential. Thus, the functional restoration of ADSCs from the aged was expected to be required before the transplantation, and the optimized strategy to improve ADSC activity would contribute to the better efficacy in vivo. 

In this study, the osteogenic potential was comparatively evaluated in ADSC-Y and ADSC-E, showing that ADSC-E has poor osteogenic differentiation potential. Therefore, ADSCs from elderly patients may have low bone-forming capacity when applied autologously. The differentiation process occurs via the binding of soluble growth factors on its receptor, emphasizing the need for sufficient growth factors and their receptors in ADSCs for the differentiation process. In other words, the paracrine potential will have implications on the extent of osteogenic differentiation.

Many growth factors/cytokines that promote osteogenesis have been reported in previous studies. Comparing the generation of soluble factors relating to osteogenesis in ADSC-Y and ADSC-E revealed that FGF2, HGF, and VEGF have a high correlation with age and osteogenic potential, not BMP-2 and TGF-β1. We gleaned that FGF2, HGF, and VEGF could be supplementary factors for ADSC-E. Previous research investigated the interactive action of FGF2, HGF, and VEGF in diverse cell types and ascertain that FGF2 or HGF could promote VEGF secretion [48,57]. Thus, this study has employed FGF2 and HGF as supplementary factors to improve the osteogenic activity of ADSC-E. 

Under an osteogenic stimulus, the expression of FGF2 and HGF was extremely low in ADSC-E compared to ADSC-Y. The FGF2 receptor expression was rarely detected in ADSC-E during the osteogenic process. ADSC-Y could have active cellular signaling for FGF2 or HGF, whereas ADSC-E had an inadequate signaling response due to the lack of ligands and receptor proteins. Indeed, the levels of Runx-2, activated by FGF2 or HGF, were rapidly elevated upon osteogenic stimulus in ADSC-Y, but osteogenic growth was slowed down in ADSC-E. This difference in Runx-2 expression is related to the osteogenic potential. This result hints that the supplementation of FGF2 and HGF could be a clue to the weak differentiation potential of ADSC-E, possibly by creating osteogenesis-favored intracellular conditions. 

To examine the enhanced osteogenic potential of ADSC-E by FGF-2 and/or HGF priming, ADSC-E was stimulated with FGF2 and/or HGF under osteogenic conditions for 6 days and then cultured for 14 days in osteogenic media. Early enrichment of FGF2 and/or HGF in ADSC-E enhanced osteogenic potential with sufficient calcium deposition. Notably, the effect of a combination of FGF2 and HGF was much better than FGF2 or HGF treatment. Priming ADSC-E with FGF2 and/or HGF for three days facilitated osteogenesis, compared to non-priming control, but its effect was less than for six days. FGF2 and/or HGF priming provoked the early shift of Runx-2 expression in ADSC-E, which might promote the osteogenic process. Additionally, the combination of FGF2 and HGF could increase the protein level of the FGF2 receptor, causing cellular status with dynamic signaling for exogenously added FGF2 and accelerating the secretion of VEGF in ADSC-E. These multiplicative effects of FGF2 and HGF are assumed to contribute to improving the osteogenic potential of ADSC-E. 

Despite the apparent effect of FGF2 and/or HGF priming on differentiation of ADSC-E in vitro, transplantation time for osteogenesis in vivo was not determined because the success of bone formation in vivo depends on the commitment of transplanted cells. Additionally, in vitro and in vivo conditions are different. The criteria to determine the ideal cellular condition for transplantation are lubricous, and its result varies depending on the research. ADSC-E was surmised to fail bone formation due to the lack of osteogenic commitment signal, and mature osteoblast is challenging to incorporate into host tissue due to its fully differentiated state. Immature osteoblast is estimated to be appropriate for transplantation, but it is difficult to decide the status of immature osteoblast in osteoinductive conditions. 

Runx-2 expression has been used as the initial factor to modulate osteogenesis. Under osteogenic induction in vitro, Runx-2 expression peaks within several days post osteogenic induction and is maintained for the proliferation of osteoblast later. 

ADSC-E with FGF2 and/or HGF priming for 3 or 6 days that may correspond to immature osteoblast was transplanted, and ectopic bone-forming capacity was examined. Histological analysis corroborates that the effect of a combination of FGF2 and HGF for 6 days are unequal in aspects of new bone structure and human osteocalcin expression. ADSCs with FGF2 or HGF treatment also could form ectopic bone, but its impact was weaker than the combination of FGF2 and HGF. The result from in vivo ectopic bone formation is consistent with the in vitro data in this study.

This study uncovers the distinct difference in paracrine potential of ADSC-Y and ADSC-E. The alteration of secretory factors is closely correlated with impaired differentiation potential of stem cells. The early priming of stem cells with FGF2 and HGF for supplementation of paracrine potential was enough to recover osteogenic potential, which occurred via the promotion of Runx-2 expression and BMP-2/VEGF secretion. Thus, ex vivo culture of ADSCs with poor cellular activity for transplantation might need extra growth factors, through which a dramatic efficacy would be achieved post-transplantation. 

This study primarily focused on ADSCs from the young and elderly. ADSCs with low osteogenic activity can be shown in various pathological situations, including acute/chronic inflammatory disease and aging. Thus, growth factor priming is expected to be broadly used to enhance the differentiation of ADSCs with low osteogenic potential and paracrine activity. Next, the efficacy of FGF2/HGF priming on the osteogenic potential of ADSCs from donors with chronic diseases will be widely evaluated, and its effect on bone defects will be confirmed using a non-clinical osteoporosis model. 

## Data Availability

The datasets used and/or analyzed during the present study are available from the corresponding author upon reasonable request.

## Supplementary Materials

The following supporting information can be downloaded at: https://www.mdpi.com/article/10.3390/cells11132042/s1, Figure S1: Effect of age on growth factor expression in ADSC, Figure S2: The effect of FGF2 priming on osteogenic potential of ADSC-E, Figure S3: The effect of FGF2 priming time on osteogenesis of ADSC-E, Figure S4: The osteogenic effect of FGF-2/HGF priming for 3 days in ADSC-E, Table S1: Characterization of human adipose-derived stem cells Figure S5: The effect of combination of FGF-2 and HGF priming on osteogenesis of ADSC-E.
