# Supplementary material for: Priming with a Combination of FGF2 and HGF Restores the Impaired Osteogenic Differentiation of Adipose-Derived Stem Cells
Source: Cells. 2022 Jun 27;11(13):2042. doi: 10.3390/cells11132042 (PMC9265418; doi:10.3390/cells11132042)
Supplement: Supplementary file 1 [file cells-11-02042-s001.zip › cells-1773767-supplementary.pdf]

Table S1

	Percentage of expression (%)	
	**Young**	**Elderly**
CD45	0.58 ± 0.02	0.13 ± 0.08
CD29	99.9 ± 0.03	99.4 ± 0.21
CD105	99.8 ± 0.08	99.9 ± 0.12
CD34	0.13 ± 0.01	0.24 ± 0.17
CD44	99.8 ± 1.2	99.2 ± 1.86
CD90	99 ± 1.2	99.5 ± 0.57

Supplementary Table S1. Characterization of human adipose derived stem cells ADSCs were analyzed by flow cytometry analysis. Fixed cells were treated with FITC-conjugated antibodies against CD45, CD29, CD105, CD34, and CD90 for 20 mins at 4°C in the dark. After washing with buffer twice, cells were re-suspended with buffer and analyzed with FACS Calibur using Cell Quest software (BD Biosciences, San Jose, CA, USA).

Figure S1

A

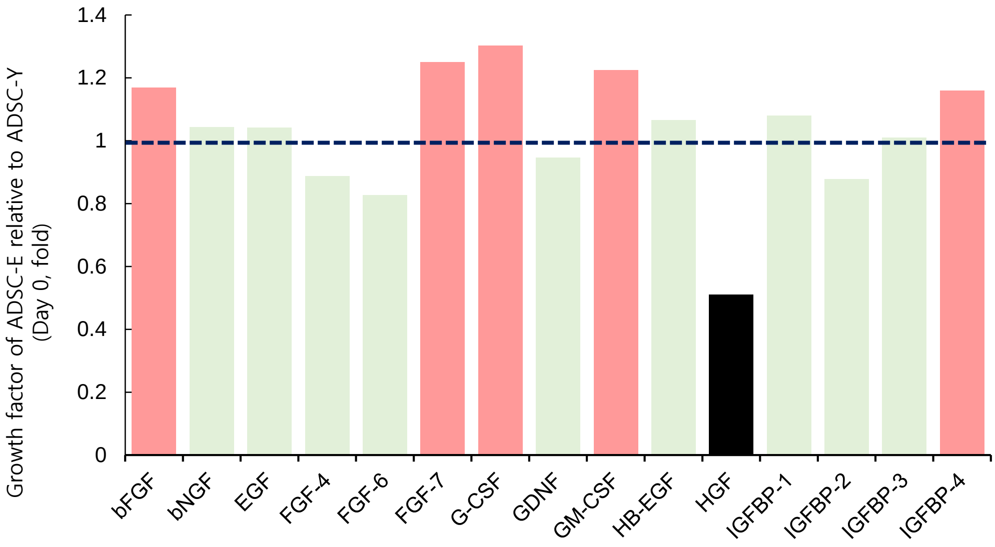

B

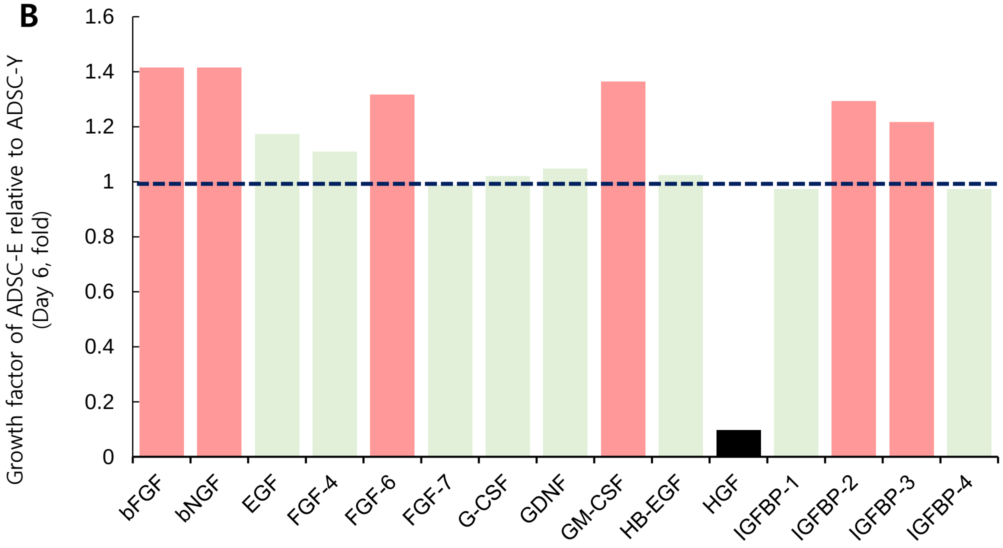

Supplementary Figure S1. Effect of Age on Growth Factor Expression in ADSC

Conditioned medium of ADSC-E and ADSC-Y was collected at day 0 and day 6 post osteogenic induction. Growth factors in each conditioned medium was analyzed with Ray Biotech Human Growth Factor Array C1 according to the manufacturer's instruction. Intensity was determined by chemiluminescence with an Amersham Imager 600. (Figure A and B) Graphs represent growth factor (fold) for ADSC-Y relative to that of ADSC-Y using Image J program. Red bar: growth factors increased in ADSC-E, comparing to ADSC-Y. Green bar: No difference between two groups or . Black bar: decreased in ADSC-E.

Figure S2

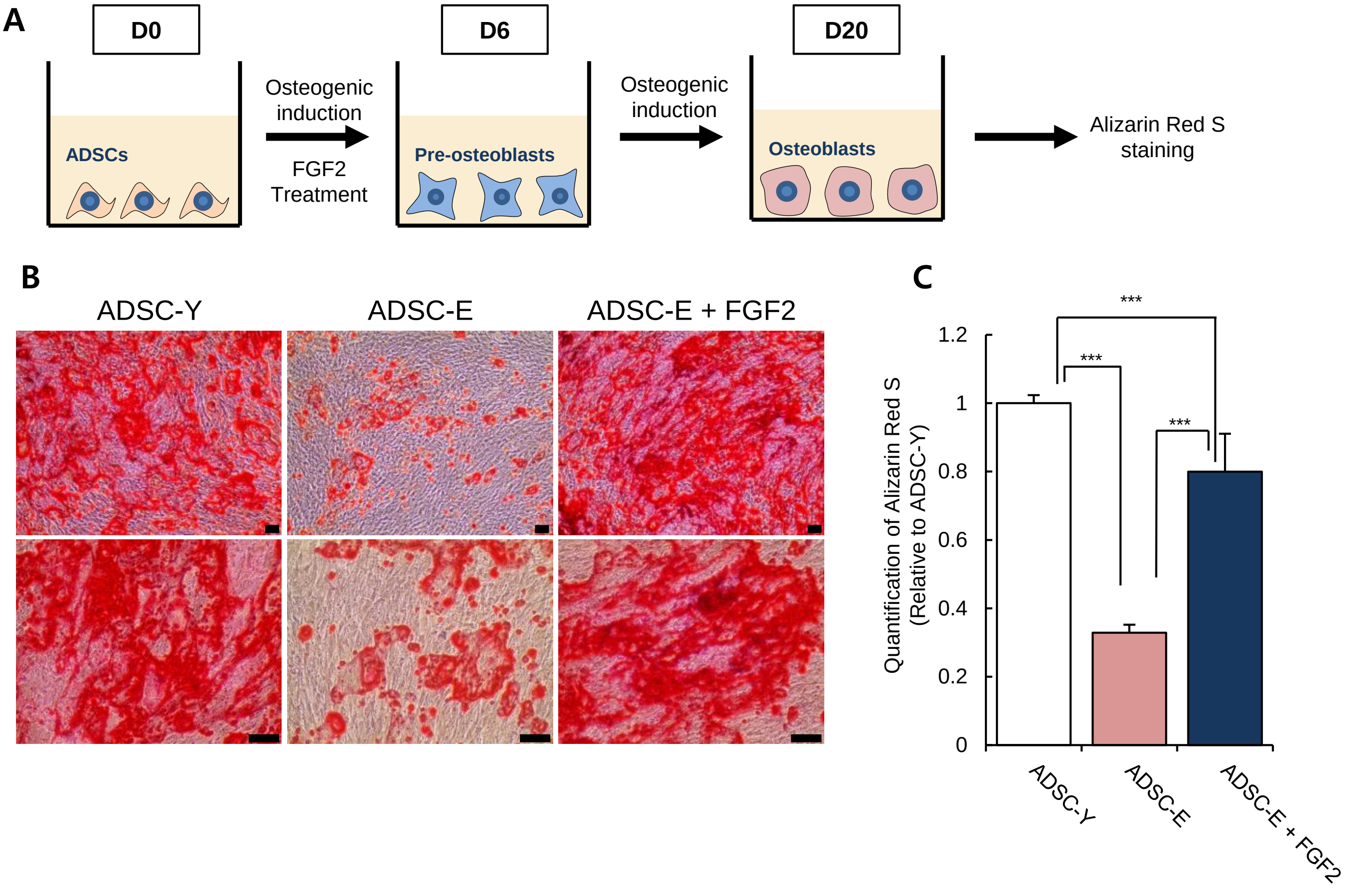

Supplementary Figure S2. The effect of FGF2 priming on osteogenic potential of ADSC-E

(A) Experimental scheme to treat FGF2 to ADSC-E during osteogenesis in vitro. (B) Representative images for Alizarin Red S staining post 20 days of osteogenic induction in ADSC-Y, ADSC-E and FGF2 primed ADSC-E. scale bar, 100 μm. (C) Quantification of Alizarin Red S relative to ADSC-Y. Results are shown as the mean ± SD of at least three replicate wells for each group (***p < 0.001)

Figure S3

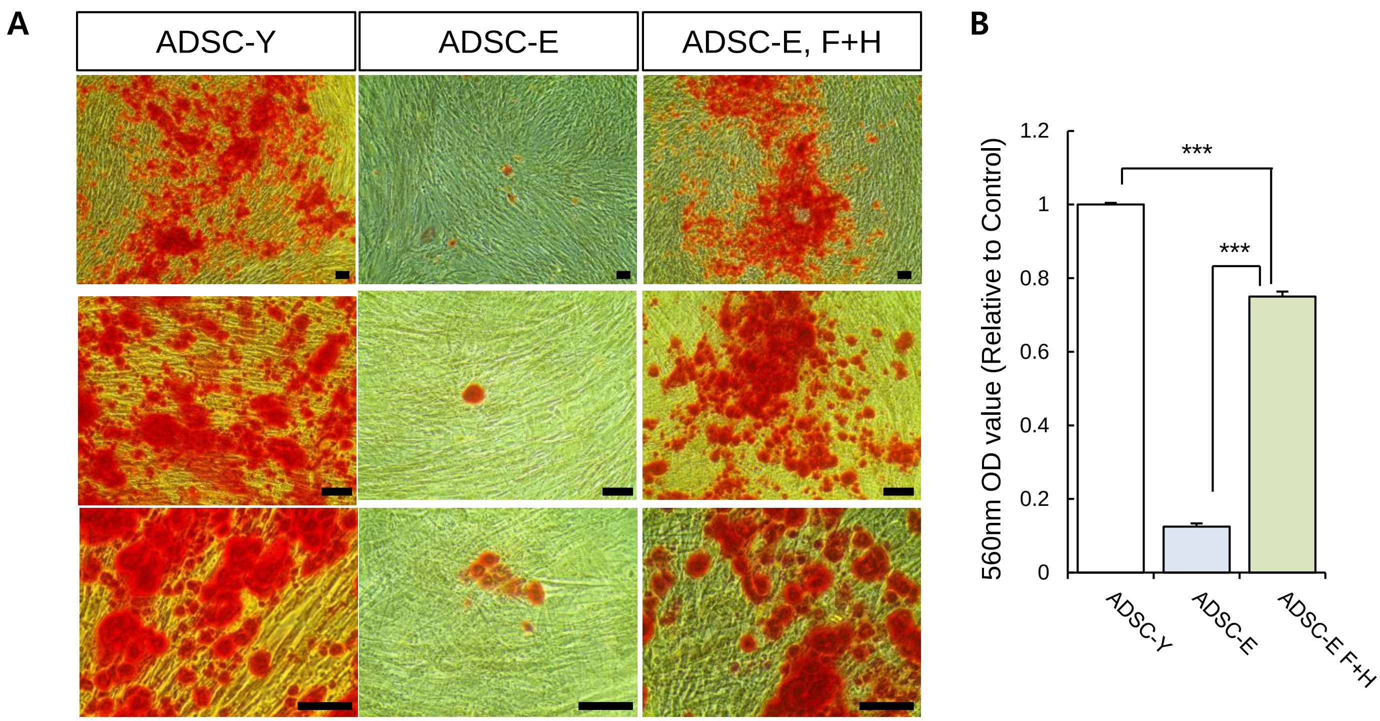

Supplementary Figure S3. The effect of combination of FGF-2 and HGF priming on osteogenesis of ADSC-E.

ADSC-E was primed with FGF2 and HGF for 6 days during osteogenic induction. At 20 days post induction, calcium deposition was observed by alizarin red staining. (A) Representative images of Alizarin Red S staining of ADSC-Y, ADSC-E and FGF2/HGF-primed ADSC-E. scale bar, 100 μm. (B) Alizarin Red S was quantified and represented relative to ADSC-Y. Results are shown as the mean ± SD of at least three replicate wells for each group (***p < 0.001)

Figure S4

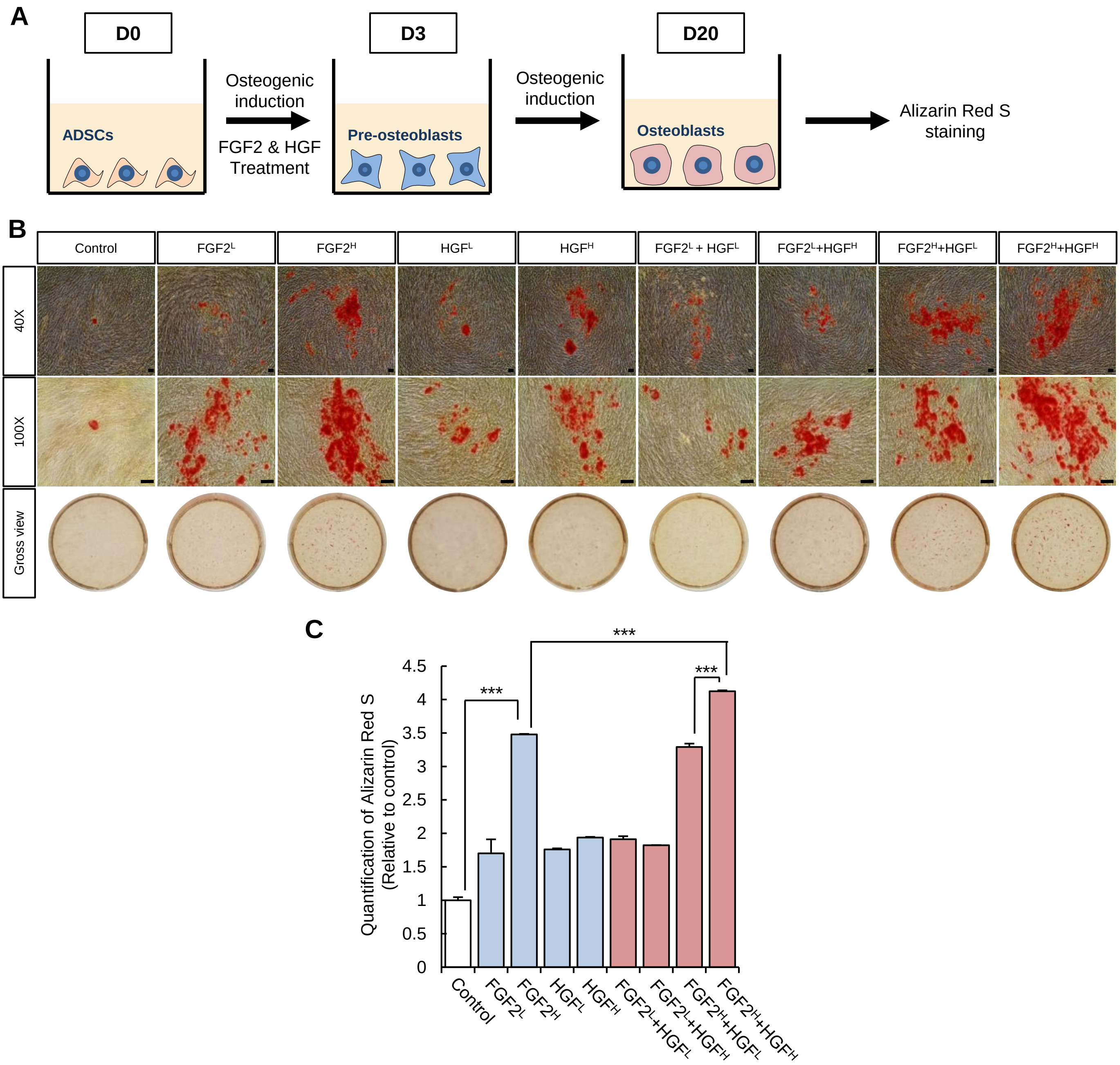

Supplementary Figure S4. The osteogenic effect of FGF-2/HGF priming for 3 days in ADSC-E

(A) Experimental design for FGF2 and/or HGF priming in ADSC-E under osteogenic induction (B) Representative images of Alizarin Red S staining after 20 days of induction of osteogenesis in ADSC-E. scale bar, 100 μm. (C) Alizarin Red S quantified with 10% (w/v) cetylpyridinium chloride was shown as a graph relative to control. Results are shown as the mean ± SD of at least three replicate wells for each group (***$p < 0.001$)

Figure S5

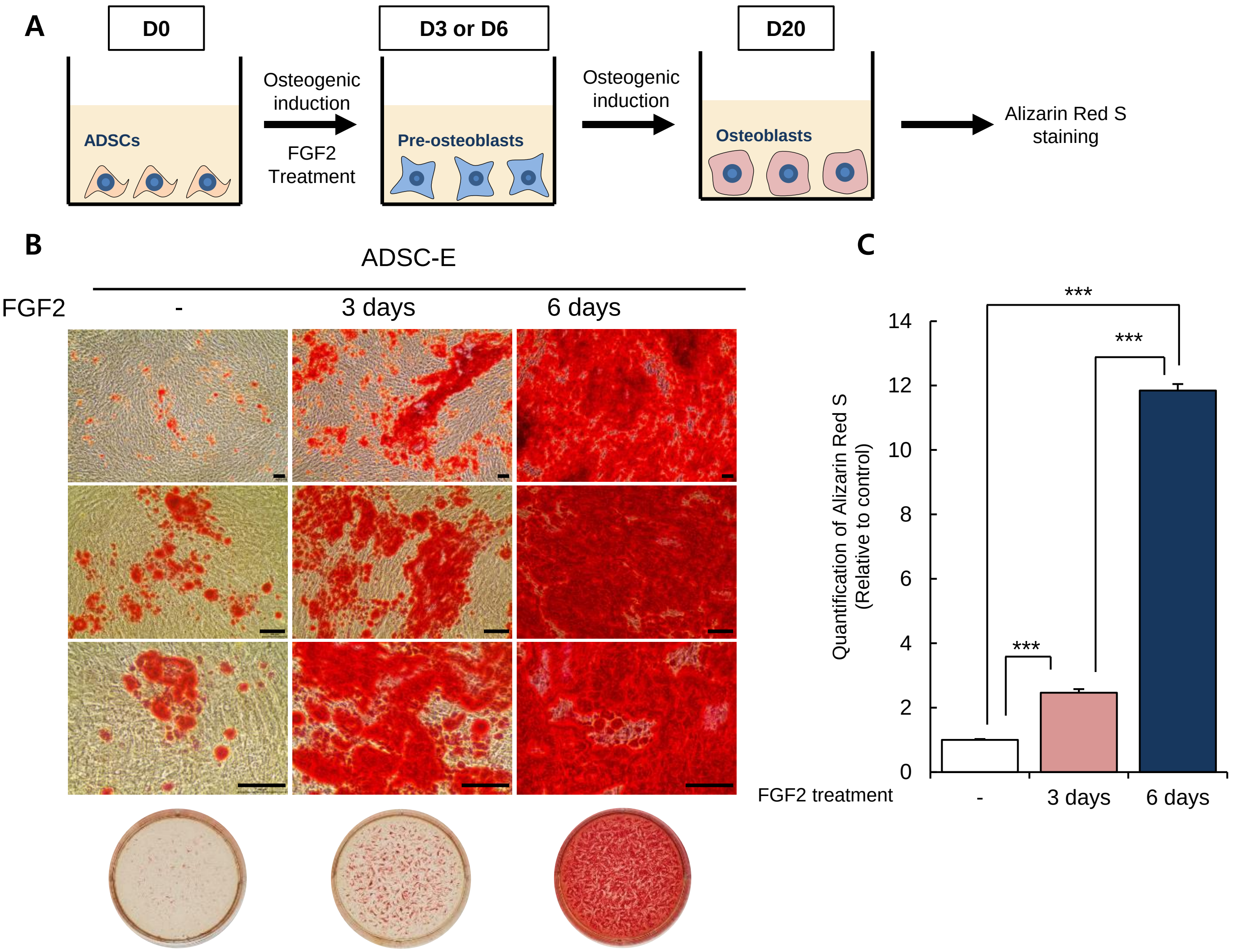

Supplementary Figure S5. The effect of FGF2 priming time on osteogenesis of ADSC-E.

(A) Experimental design for treatment of FGF2 to ADSC-E during osteogenic induction. (B) Representative images of Alizarin Red S staining after 20 days of osteogenic induction in ADSC-Y and FGF2-primed ADSC-E for 3 or 6 days. scale bar, 100 μm. (C) ADSC-E primed with FGF2 for 3 or 6 days was quantified relative to the control. Results are shown as the mean ± SD of at least three replicate wells for each group (***p < 0.001)
